# Discovery of a novel type IIb RelBE toxin‐antitoxin system in *Mycobacterium tuberculosis* defined by co‐regulation with an antisense RNA

**DOI:** 10.1111/mmi.14917

**Published:** 2022-05-24

**Authors:** Clinton C. Dawson, Jason E. Cummings, Julie M. Starkey, Richard A. Slayden

**Affiliations:** ^1^ Mycobacteria Research Laboratories, Department of Microbiology, Immunology, and Pathology Colorado State University Fort Collins Colorado USA; ^2^ Present address: Endolytix Technology Beverly MA USA

**Keywords:** antisense RNA, double‐stranded RNase III dependent decay, *Mycobacterium tuberculosis* noncoding RNA, toxin‐antitoxin regulation, toxin‐antitoxin systems

## Abstract

Toxin‐antitoxin loci regulate adaptive responses to stresses associated with the host environment and drug exposure. Phylogenomic studies have shown that *Mycobacterium tuberculosis* encodes a naturally expanded type II toxin‐antitoxin system, including ParDE/RelBE superfamily members. Type II toxins are presumably regulated exclusively through protein–protein interactions with type II antitoxins. However, experimental observations in *M. tuberculosis* indicated that additional control mechanisms regulate RelBE2 type II loci under host‐associated stress conditions. Herein, we describe for the first time a novel antisense RNA, termed asRelE2, that co‐regulates RelE2 production via targeted processing by the *Mtb* RNase III, Rnc. We find that convergent expression of this coding‐antisense hybrid TA locus, *relBE2‐asrelE2*, is controlled in a cAMP‐dependent manner by the essential cAMP receptor protein transcription factor, Crp, in response to the host‐associated stresses of low pH and nutrient limitation. Ex vivo survival studies with *relE2* and *asrelE2* knockout strains showed that RelE2 contributes to *Mtb* survival in activated macrophages and low pH to nutrient limitation. To our knowledge, this is the first report of a novel tripartite type IIb TA loci and antisense post‐transcriptional regulation of a type II TA loci.

## Graphical abstract

Our investigation uncovered a novel antisense RNA, asRelE2, that regulates *relE2* in the type II *relBE2* locus. The transcription of this novel tripartite hybrid type IIb TA locus, *relBE2‐asrelE2*, is regulated by Crp_mtb_ in a cAMP‐dependent manner. RelB2 and RelE2 are differentially regulated by targeted degradation of the *relE2‐asrelE2* dsRNA hybrid by the RNases, Rnc. This novel molecular mechanism ultimately implicates antisense‐mediated differential regulation of TA systems in *Mtb* persistence and pathogenesis.

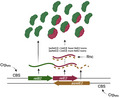

## 1 INTRODUCTION

Despite more than 60 years of available treatments, multidrug regimens, and disease management strategies, *Mycobacterium tuberculosis* (*Mtb*), the causative agent of tuberculosis (TB), remains one of the most medically important pathogens (WHO, 2021). It is widely accepted that a significant proportion of the world's population has been exposed to *Mtb*. While most individuals either do not become infected or effectively control the infection long‐term (Demissie et al., 2004), 5%–10% of these individuals will develop active TB disease in their lifetime and, thus, represent a significant source of new infections worldwide (Fox et al., 2017; Wood et al., 2011). The overall inability to control tuberculosis has been associated with the required lengthy multidrug regimens that often fail to effectively target all members of the phenotypically diverse bacterial populations and tolerant tubercle bacilli, resulting in latent tuberculosis infection (LTBI) that is refractory to treatment (https://www.niaid.nih.gov/diseases‐conditions/tbdrugs). *Mtb* is known to establish an adaptive persistent state in a host‐activated immune environment, which is critical for establishing and maintaining a chronic *Mtb* infection (Dutta et al., 2010; Mariotti et al., 2013).

Studies have shown that *Mtb* type II toxin‐antitoxin (TA) systems are significantly and differentially regulated in response to host‐associated stresses encountered by tubercle bacilli during infection, implying that TA loci are involved in establishing and maintaining a persistent state (Agarwal et al., 2018, 2020; Gupta et al., 2017; Keren et al., 2011; Korch et al., 2009; Ramirez et al., 2013; Singh et al., 2010; Tiwari et al., 2015). It is presumed that during periods of active growth, such as the acute phase of *Mtb* infection, the transcription and translation of type II toxins and antitoxins are tightly coupled to ensure the production of equivalent stoichiometries of the toxin and its neutralizing antitoxin (Ramirez et al., 2013; Singh et al., 2010; Slayden et al., 2018; Tandon et al., 2019). However, under stressful conditions, such as host immune environments encountered by *Mtb* during chronic infection, cognate antitoxin and toxin protein levels are thought to be dysregulated by targeted degradation of the antitoxin, thereby freeing toxins (Yamaguchi et al., 2011). Accordingly, when the protein toxins become more abundant than the protein antitoxins, they induce bacteriostasis by cleaving translating mRNAs engaged with the ribosome, resulting in ribosomal stalling on truncated messages (Neubauer et al., 2009). This results in a transition from acute growth to a nonreplicating persistent state (NRP) characteristic of treatment tolerant infections involving bacterial adaptive responses and alternative and reduced metabolism (Ramirez et al., 2013). However, the regulation and coordination of the majority of *Mtb* TA loci are not defined.

A growing body of evidence has indicated that post‐transcriptional regulation, including antisense transcription, which has been reported to be extensive in *Mtb*, is a hallmark of bacterial pathogenesis (Arnvig & Young, 2009; DiChiara et al., 2010; Dinan et al., 2014; Sesto et al., 2013). *Mtb* is known to transcribe complementary RNAs to approximately two‐thirds of its annotated open reading frames (ORFs) during the exponential phase and more than 90% in the stationary phase (Arnvig et al., 2011). Such large numbers of antisense (as)RNAs are thought to modulate gene expression primarily and protein production levels by double‐stranded (ds) RNA‐dependent decay via the ribonuclease III protein, RNase III (Lasa et al., 2011). This has been further substantiated by specific reports that antisense regulation leads to a differential abundance of genes that are co‐transcribed in polycistronic messages essential to the virulence (Arnvig et al., 2011; Arnvig & Young, 2009; DiChiara et al., 2010; Matsunaga et al., 2004; Movahedzadeh et al., 2004; Schnappinger et al., 2003). Interestingly, we have repeatedly observed significant differences in the abundance of type II cognate antitoxin and toxin mRNAs, including *relB2* and *relE2*, under stress conditions that are presumably co‐expressed as part of a single bicistron leading us to believe that select *Mtb* TA loci are post‐transcriptionally regulated as part of broader adaptive responses to the host environment and immune stresses (Ramirez et al., 2013; Slayden et al., 2018).

Our investigation uncovered a novel antisense RNA asRelE2 encoded by *ncRv2866Ac* on the complementary strand of the type II *relBE2* locus (Rv2865‐Rv2866 or RelFG). We determined that convergent transcription of this novel tripartite hybrid type II TA locus, *relBE2*‐*asrelE2*, is regulated by the essential stress‐responsive transcription factor cAMP receptor protein, Crp_,_ in a cAMP‐dependent manner. Under host‐associated environments such as low pH and nutrient limitation, we found that *relE2* mRNA expression levels were significantly and differentially upregulated relative to *relB2* and contrary to asRelE2. Ex vivo survival studies with *relE2* and *asrelE2* knockout strains showed that asRelE2 regulates RelE2, and RelE2 contributes to *Mtb* survival to low pH and nutrient limitation and activated macrophages (Mϕs). To our knowledge, this is the first report of a unique tripartite type II TA locus we have termed a type IIb defined by co‐regulation by the cognate antitoxin protein and antisense RNA to the toxin. This novel molecular mechanism ultimately implicates antisense‐mediated differential regulation of TA systems in *Mtb* persistence and pathogenesis.

## 2 RESULTS

### 2.1 Identification and mapping of a novel cis‐encoded antisense RNA, asRelE2

**FIGURE 1:**
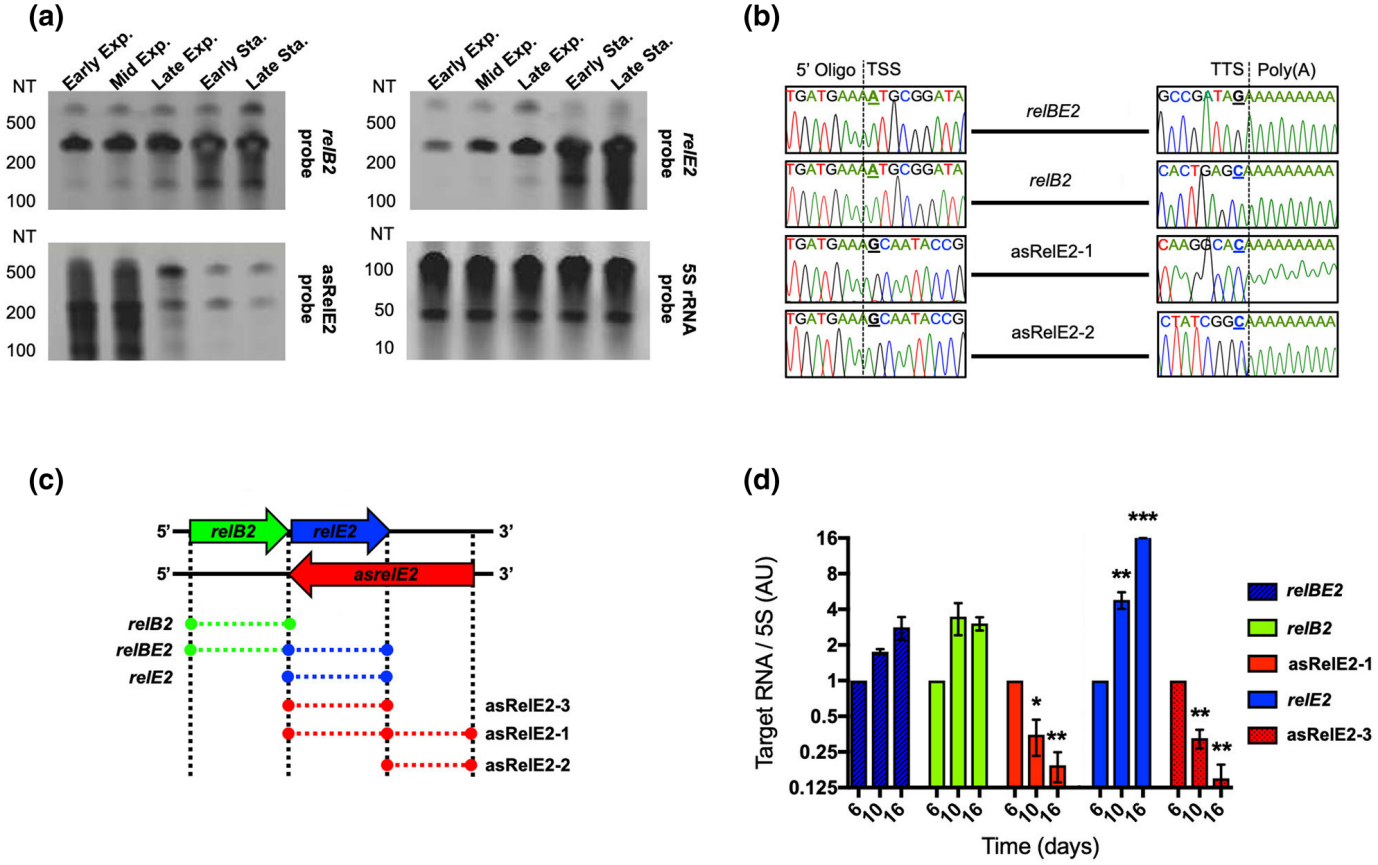
Characterization of the antisense, asRelE2. (a) Northern blots of total RNA isolated from WT *Mtb* probing against *relE2*, *relB2*, asRelE2, and 5S rRNA. (b) Consensus mapping of 5′ and 3′ ends of *relBE2* and asRelE2 primary transcripts. (c) Schematic of the genomic context of the *relBE2 asrelE2* TA locus encoded in WT *Mtb* str. H37Rv and transcripts. (d) Relative quantification of *relBE2* and asRelE2 transcript. WT *Mtb relBE2, relB2, relE2*, asRelE2‐1, and asRelE2‐3 transcript levels were quantified by densitometry, normalizing target transcripts to corresponding 5S rRNA blots (AU), similarly as previously reported by Muller et al. (2016). Relative to day 6, significant differences were assessed using a two‐way ANOVA with Tukey's multiple comparisons post‐tests (*p‐value <0.05; **p‐value <0.01; ***p‐value <0.001).

By definition, type II toxins are encoded in bicistrons and regulated exclusively at the post‐transcriptional level through protein–protein interactions with the type II antitoxins (Korch et al., 2009; Miallau et al., 2013; Riffaud et al., 2020; Wessner et al., 2015). Interestingly, we have repeatedly observed significant differences in the abundance of *Mtb* type II cognate antitoxin and toxin mRNAs, including *relB2* and *relE2*, co‐expressed in a single bicistron. This observation indicates an additional mechanism of regulation, likely at the post‐transcriptional level, that alters the relative mRNA abundances of the *relE2* toxin relative to the *relB2* antitoxin. One common mechanism of post‐transcriptional regulation of mRNA abundance involves antisense RNA (Sesto et al., 2013). Therefore, we investigated the presence of an asRNA as a possible co‐regulatory mechanism that differentially controls *relB2* or *relE2* mRNA levels. Northern blot analysis was performed with total RNA isolated from *Mtb* at different growth phases using riboprobes designed to identify sense and antisense *relBE2* transcripts (Figure 1a). The *relB2*‐specific riboprobe identified 282‐nucleotide (NT) and 549‐NT fragments corresponding to *relB2* and *relBE2* mRNAs. The *relE2*‐specific riboprobe identified 264‐NT and 549‐NT length fragments corresponding to *relE2* and *relBE2* mRNAs. Notably, two novel RNAs, 512‐NT and 264‐NT in size, corresponding to *asrelE2‐1* and *asrelE2‐2*, were also discovered.

Rapid amplification of cDNA ends (RACE) was applied to map the identified fragments using sense and antisense *relB2‐*specific and *relE2*‐specific riboprobes. Sequencing of the 5′/3′ RLM‐RACE PCR products mapped the transcriptional start sites (TSSs) of the 282‐NT and 549‐NT fragments corresponding to *relB2* and *relBE2* to genome base A‐3177537, the first NT in the start codon of *relB2*, and the 3′ ends to genome bases C‐3177820 and G‐3178085, the third NT in the stop codon of *relB2* and *relE2*, respectively (Figure 1b). Sequencing the 5′/3′ RLM‐RACE products mapped the 5′ and 3′ ends of the 512‐NT fragment of *asrelE2‐1* to genome bases G‐3178333 and C‐3177822, respectively. Sequencing the 5′/3′ RLM‐RACE products amplified from enriched cleaved RNAs containing 5′ monophosphorylated (PO_4_) ends revealed that asRelE2‐3 fully complements *relE2* and is processed from the primary transcript asRelE2‐1 (Figure 1c). Sequencing of additional clones of the 5′/3′ RACE PCR products with 5′ monophosphorylated RNAs identified 6‐NTs directly upstream (5′ UGAGCG 3′) as the consensus 5′ end of processed *relE2* mRNA, along with the corresponding 6‐NTs on the complementary strand as the consensus 3′ end of asRelE2‐2 (Figure 1c).

### 2.2 RelE2 is co‐regulated by asRelE2‐1, asRelE2‐3, and the RelB2 antitoxin

Monitoring *relE2* and asRelE2 expression after 6, 10, and 16 days of growth revealed that *relE2* mRNA increased more than 20‐fold relative to the constitutively expressed 5S rRNA (MTB00002 or Rrf), which is consistent with the greater abundance of *relE2* in later growth phases observed via northern blotting (Figure 1a,d). In contrast, asRelE2‐1 and asRelE2‐3 declined sharply over time, decreasing by 20‐fold in the stationary phase. Concomitant constitutive expression of *relBE2* and *relB2* mRNA levels were observed throughout in vitro growth, increasing only 2‐ to 4‐fold over the same period (Figure 1d). These findings indicate that asRelE2‐1 is processed, resulting in asRelE2‐3, which directly interacts and differentially modulates *relE*2 mRNA expression levels in a growth phase‐dependent manner.

**FIGURE 2:**
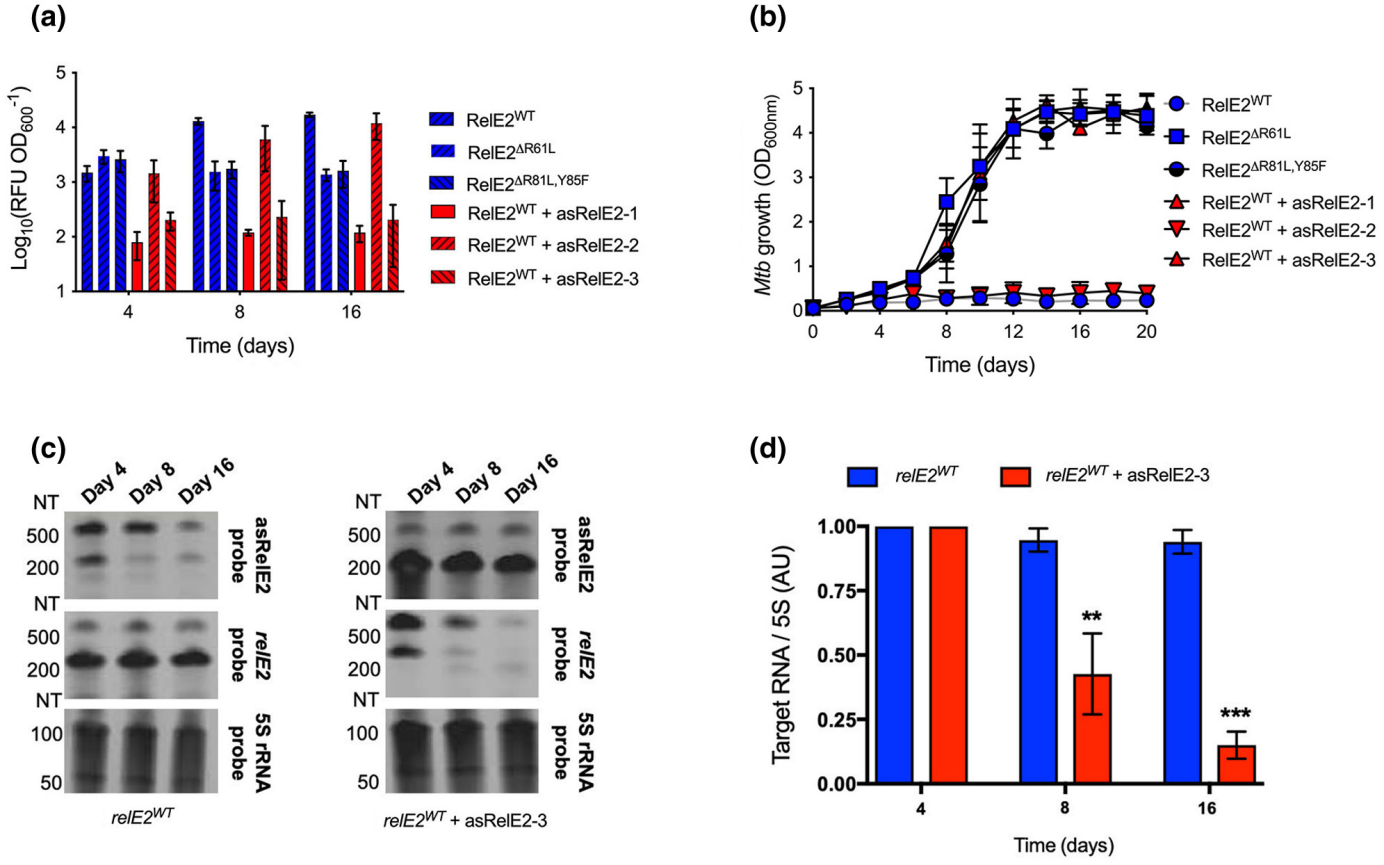
asRelE2‐1 and asRelE 2–3 silence *relE2* translation in situ. (a) Production of wildtype and mutant RelE2 proteins alone and when co‐expressed with asRelE2‐1 and the processed transcripts asRelE2‐2 and asRelE2‐3. (b) Corresponding growth of *Mtb* over a 20‐day experimental period. (c) Northern blots of total RNA isolated from *Mtb* strains overexpressing WT *relE2* and WT *relE2* with asRelE2. (d) Relative quantification using densitometry. Relative to day 4, significant differences were assessed using a two‐way ANOVA with Tukey's multiple comparisons post‐tests (**p‐value <0.01; ***p‐value <0.001).

To determine how asRelE2 targets and silences RelE2 production in situ, we characterized the co‐overexpression of *asrelE2‐1*, *asrelE2‐2*, and *asrelE2‐3* on RelE2 production using a tandem ATc‐inducible P_myc_tetO fluorescent protein overexpression system. Nonfunctional RelE2 mutants, RelE2^ΔR61L^, and RelE2^ΔR81L,Y85F^ with amino acid (AA) substitutions at arginine‐(R)61, or at R81, and tyrosine‐(Y)85 corresponding to essential catalytic residues in prototype *E. coli* RelE were engineered and utilized as functionally inactive positive production controls (Neubauer et al., 2009). Induction of WT *relE2*, *relE2* ^*ΔR61L*^, or *relE2* ^*ΔR81L,Y85F*^ alone resulted in protein production as indicated by fluorescence units (RFUs) over time in situ (Figure 2a). In contrast, no increase in RFUs was observed with co‐overexpression of *relE2* and *asrelE2‐1* or *asrelE2‐3*, demonstrating that the full‐length primary transcript, asRelE2‐1, and the processed transcript, asRelE2‐3, directly inhibit the production of RelE2 (Figure 2a). In contrast, the *RelE2* noncomplementary asRelE2‐2 did not prevent RelE2 production, further supporting the direct inhibition of *relE2* translation by asRelE2‐1 or asRelE2‐3.

Complimentary co‐expression and growth studies assessed the ability of asRelE2‐1 or asRelE2‐3 to rescue the growth inhibition phenotype seen with RelE2 production. As expected, the expression of WT *relE2* alone inhibited *Mtb* growth (Figure 2b). In contrast, the expression of the inactivate mutants, *relE2* ^*ΔR61L*^ and *relE2* ^*ΔR81L,Y85F*^, did not affect the growth over the 20 days (Figure 2b). Similarly, when *asrelE2‐1* or *asrelE2‐3* were co‐overexpressed with WT *relE2*, logarithmic growth is comparable to the *relE2* mutants. Again, asRelE2‐2 had no regulatory effect on *relE2* based on the observation that co‐overexpression did not reverse the growth inhibition observed with WT *relE2* alone. These observations were further supported by northern blotting of *relE2* and the complementary *asrelE2‐3*, which revealed that *relE2* is downregulated ~100‐fold by co‐overexpression of *asrelE2‐3* (Figure 2c,d). These findings indicate that asRelE2 functions to silence *relE2* translation in situ and that the inhibition of RelE2 production requires complementary base‐pairing between *relE2* and asRelE2‐1 or asRelE2‐3. An in silico analysis further supports our findings, which predicts that the complementary portion present in asRelE2‐1 and asRelE2‐2 interact with *relE2* (i.e., ΔG = −226.525 kcal mole^−1^ and *p*‐value <0.0001).

Although RelBE2 has been annotated and reported as a type II TA loci, we sought to confirm the functionality and physical interaction of RelB2 and RelE2 in *Mtb* (Yang et al., 2010). When *relE2* is expressed, it induces a bacteriostatic phenotype (Figure 2b, Figure S1a). The observed bacteriostatic phenotype caused by *relE2* expression can be rescued by co‐expression of *relB2*, demonstrating a functional interaction of the cognate toxin and antitoxin proteins in vivo (Figure S1b). Co‐purification and western blotting were performed to visualize direct physical interaction between RelB2 and RelE2 (Figure S1c). When recombinant HIS‐RelB2 and RelE2‐HSV were produced individually and subjected to metal affinity chromatography, the HIS‐RelB2 was found in the bound fraction, and RelE2‐HSV was found in the unbound eluate, indicating it was not retained on the affinity column. When RelE2‐HSV was co‐overproduced with HIS‐RelB2, RelE2‐HSV was retained and co‐eluted with HIS‐RelB2, substantiating that these cognate antitoxin‐toxin proteins physically interact in situ, confirming that RelBE2 functions as a bona fide type II TA loci in *Mtb*. Together, these results indicate that the type II *relBE2* loci are co‐regulated by an antisense mechanism in addition to cognate protein–protein interactions that define type II TA systems.

### 2.3 Complementary asRelE2 mediates Rnc‐dependent decay of relE2 mRNA in vitro

**FIGURE 3:**
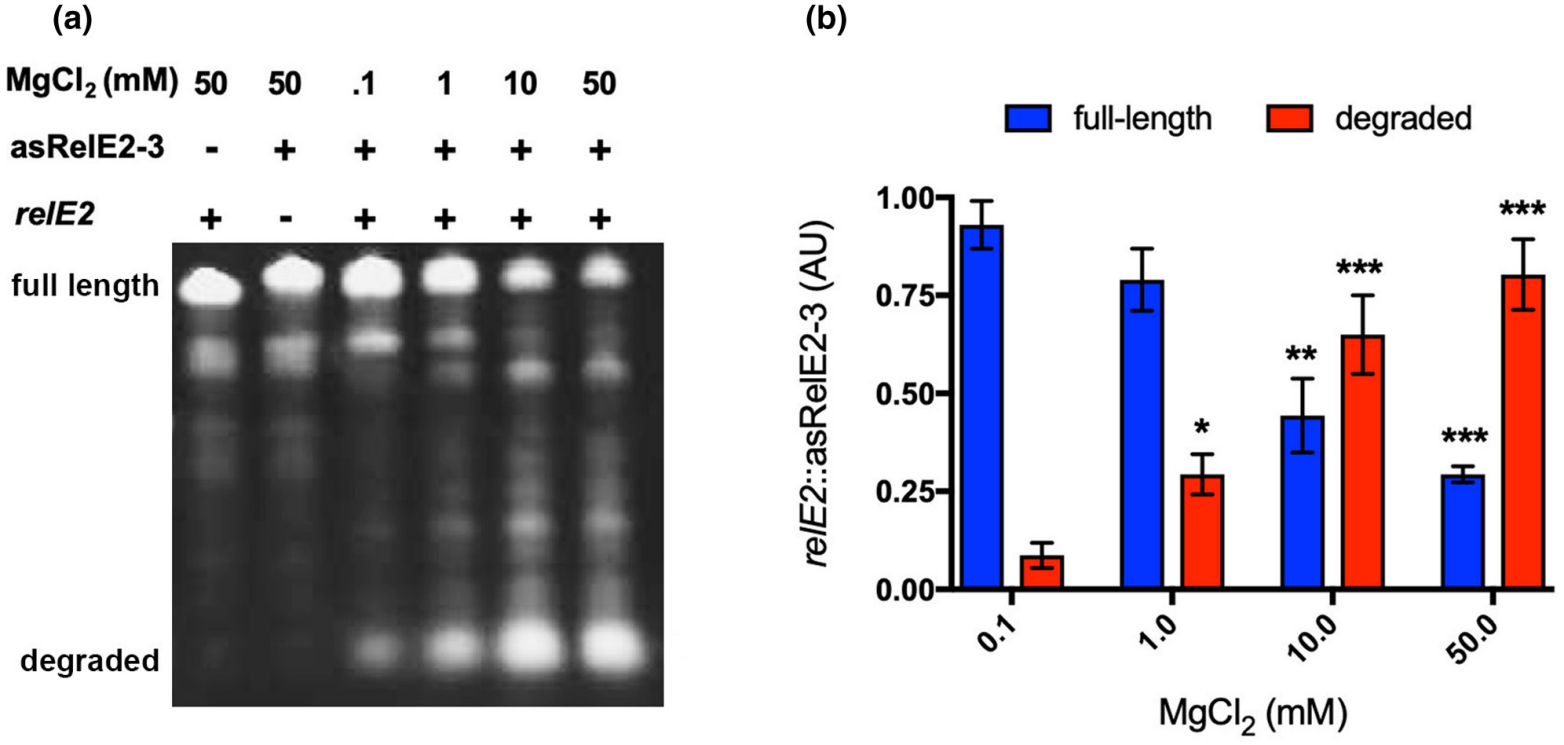
Rnc_*Mtb*_ dependent decay of *relE2*::asRelE2‐3 dsRNA. (a) Decay of in vitro transcribed *relE2*::asRelE2‐3 dsRNA hybrids by Rnc_*Mtb*_. (b) Rnc_Mtb_ RNase III activity on *relE2*::asRelE2‐3 dsRNA. All conditions contained purified Rnc_Mtb_ and relative amounts (AU) of full‐length substrates and degradation products were normalized to 0.10 mM MgCl_2_ dsRNA decay reactions and significant differences were assessed using a two‐way ANOVA with Tukey's multiple comparisons post‐tests (*p‐value <0.05; **p‐value <0.01; ***p‐value <0.001).

To determine if *relE2* is degraded by the *Mtb* RNase III, Rnc, in an asRelE2‐dependent manner, *relE2* and asRelE2‐3 in vitro transcribed RNAs were incubated in the presence of purified recombinant *Mtb* HIS‐Rnc (Figure 3a). Negative control reactions containing either full‐length *relE2* or asRelE2‐3 with HIS‐Rnc showed no degraded product. When *relE2* and asRelE2‐3 were incubated together, full‐length *relE2* and *asRelE2‐3* were found to decrease concomitantly with the apparent appearance and accumulation of degraded low molecular weight RNA products. Moreover, these decay products increased in a magnesium activation‐dependent manner characteristic of Rnc, with ~75% of the corresponding full‐length RNA species being degraded by HIS‐Rnc in 50 mM MgCl_2_ (Figure 3b). This observed Rnc‐dependent decay of *relE2* mediated by the complimentary asRelE2‐3 demonstrates that *relE2* undergoes targeted degradation by Rnc in an asRelE2‐dependent manner.

### 2.4 Crp, relBE2, and asrelE2 are differentially regulated under low pH and nutrient limitation in a cAMP‐dependent manner

**FIGURE 4:**
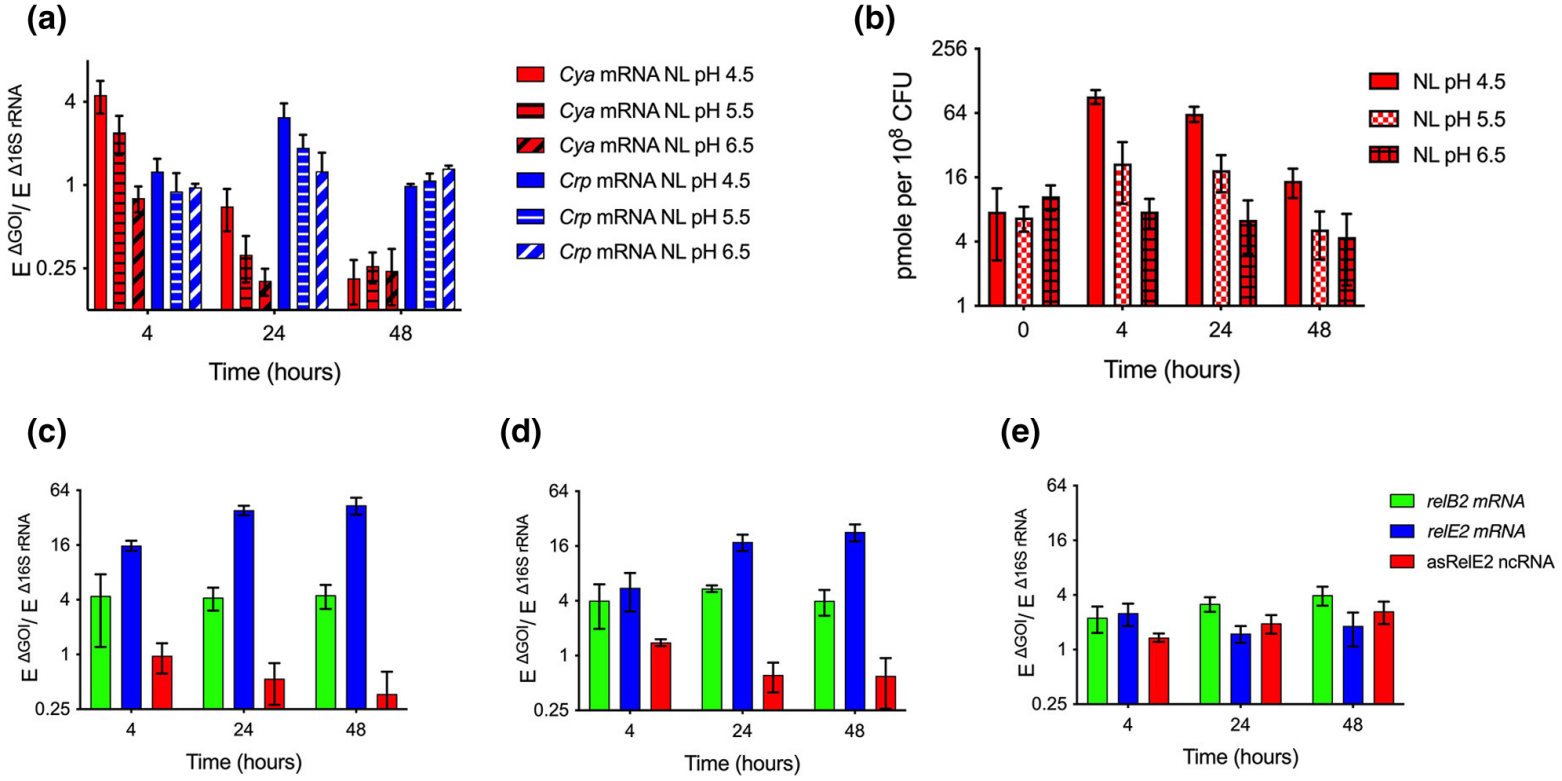
Differential regulation of *Mtb relBE2*/asrelE2 expression under defined in vitro growth conditions. (a) Gene expression analysis of adenylyl cyclase *cya* and *Crp* at low pH and nutrient limitation (NL). (b) cAMP levels at low pH and nutrient limitation (NL). Expression analysis of *relB2*, *relE2*, and asRelE2 following exposure to (c) pH 4.5, (d) pH 5.5, and (e) pH 6.5. Plotted data represent the mean, ± standard deviation of (*N* = 3) separate experiments, and significant differences were assessed using a two‐way ANOVA with Tukey's multiple comparisons post‐tests (*p‐value <0.05; **p‐value <0.01).

To assess the expression of *Crp, relBE2*, and *asrelE2* under host‐associated conditions, *Mtb* was exposed to pH 4.5 and 5.5 and nutrient‐limitation (NL). The transcription of *Crp* was transiently upregulated at 24 h of exposure to pH 4.5 and NL (Figure 4a). Similarly, *relB2 and relE2* expression increased within 24 h of exposure to pH 4.5 or pH 5.5 and NL (Figure 4c–e). In contrast, *asRelE2* was repressed at pH 4.5 or pH 5.5 and NL throughout the 48 test period (Figure 4c–e). Notably, the increased but differential expression between *relB2 and relE2* correlated with the observed decreased expression of *asRelE2*. The known pH‐responsive adenylyl cyclase (cya) transcriptional response steadily decreased to steady‐state levels within 48 h (Figure 4a). Quantitation of total cAMP confirmed that intracellular cAMP levels peaked at 80 and 20 pmol at pH 4.5 and 5.5, respectively, and remained elevated for 24 to 48 h compared to cAMP levels at pH 6.5 (Figure 4b). This analysis revealed that *Crp*, *relB2*, *relE2*, and *asrelE2* are regulated in response to the host‐associated stresses of acidic pH and nutrient limitation, correlating with altered cAMP levels.

**FIGURE 5:**
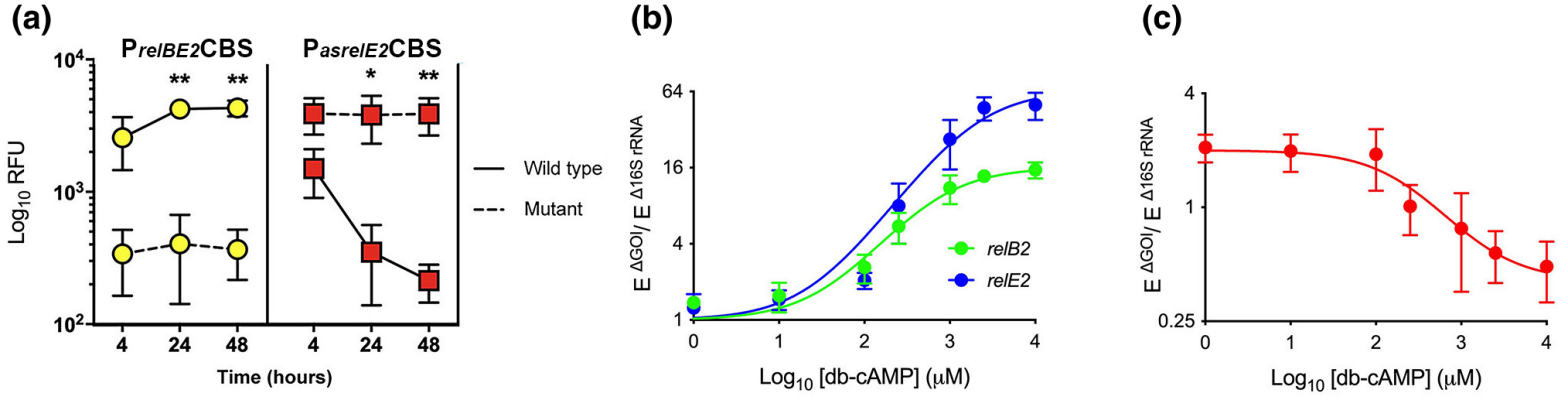
cAMP‐dependent differential regulation of RelB2, RelE2, and asRelE2. (a) Differential regulation between WT and Mut P_*relBE2*_CBS (yellow circles) and WT and Mut P_*asrelE2*_CBS (red squares) in response to intracellular cAMP. Straight lines and dashed lines profile WT and Mut P_*relBE2*_CBS and P_*asrelE2*_CBS activities in response to elevated intracellular db‐cAMP. Expression intracellular cAMP dose–response curves for (b) *relB2* and *relE2* and (c) asRelE2. RT‐qPCR gene expression analyses of total RNA isolated from mid‐exponential phase *Mtb*. Relative changes in gene expression were calculated using the E^−ΔΔCt^ method, normalized to 16S rRNA, and compared to 0 h controls. Significance determined using a regular two‐way ANOVA with Tukey's post‐tests (*p‐value <0.05; **p‐value <0.01).

A dual transcriptional reporter was engineered to examine further the cAMP‐dependent transcription of relBE2 and asrelE2 and regulation by Crp. This dual reporter was constructed with unstable *gfp* and *mcherry* variants that are transcriptionally controlled by the 105‐NTs and 120‐NTs, including the CBSs upstream IGRs of *relBE2* and *asrelE2*, respectively. Site‐directed mutagenesis was utilized to change the WT P_*relBE2*_CBS (tGAGacgccgcgCACa) and the WT P_*asrelE2*_CBS (cGACgtcctgtgCACg) to create noninducible mutant P_*relBE2*_CBS (tGGAacgccgcgCACa) and mutant P_*asrelE2*_CBS (cGCAgtcctgtgCACg) controls for direct comparison. Midexponential phase recombinant *Mtb* H37Rv cultures were exposed to dibutyryl (db)‐cAMP for 48 h. GFP RFUs driven from the WT P_*relBE2*_CBS increased significantly by approximately 10‐fold at 24 h and 40‐fold at 48 h (Figure 5a). In contrast, mCHERRY RFUs from the WT P_*asrelE2*_CBS decreased 10‐fold and 20‐fold at 24 and 48 h, respectively. To further assess the complexities of cAMP‐dependent regulation of *relBE2‐asrelE2* transcription in situ, changes in *relB2*, *relE2*, and *asrelE2* expression levels were evaluated in tandem in WT *Mtb* cultures exposed to increasing amounts of db‐cAMP for 4 h using RT‐qPCR DGE analysis. This quantitative analysis showed that *relB2* and *relE2* transcripts were regulated in a dose‐dependent manner following exposure to 100‐to‐10,000 μM of db‐cAMP. Specifically, *relB2* and *relE2* were increased 5–10‐fold and 10–50‐fold, respectively (Figure 5b). In contrast, *asrelE2* expression levels slightly decreased with increasing intracellular cAMP concentrations (Figure 5c).

### 2.5 RelE2 contributes to survival due to limited nutrient and low pH exposure and activated macrophages

**FIGURE 6:**
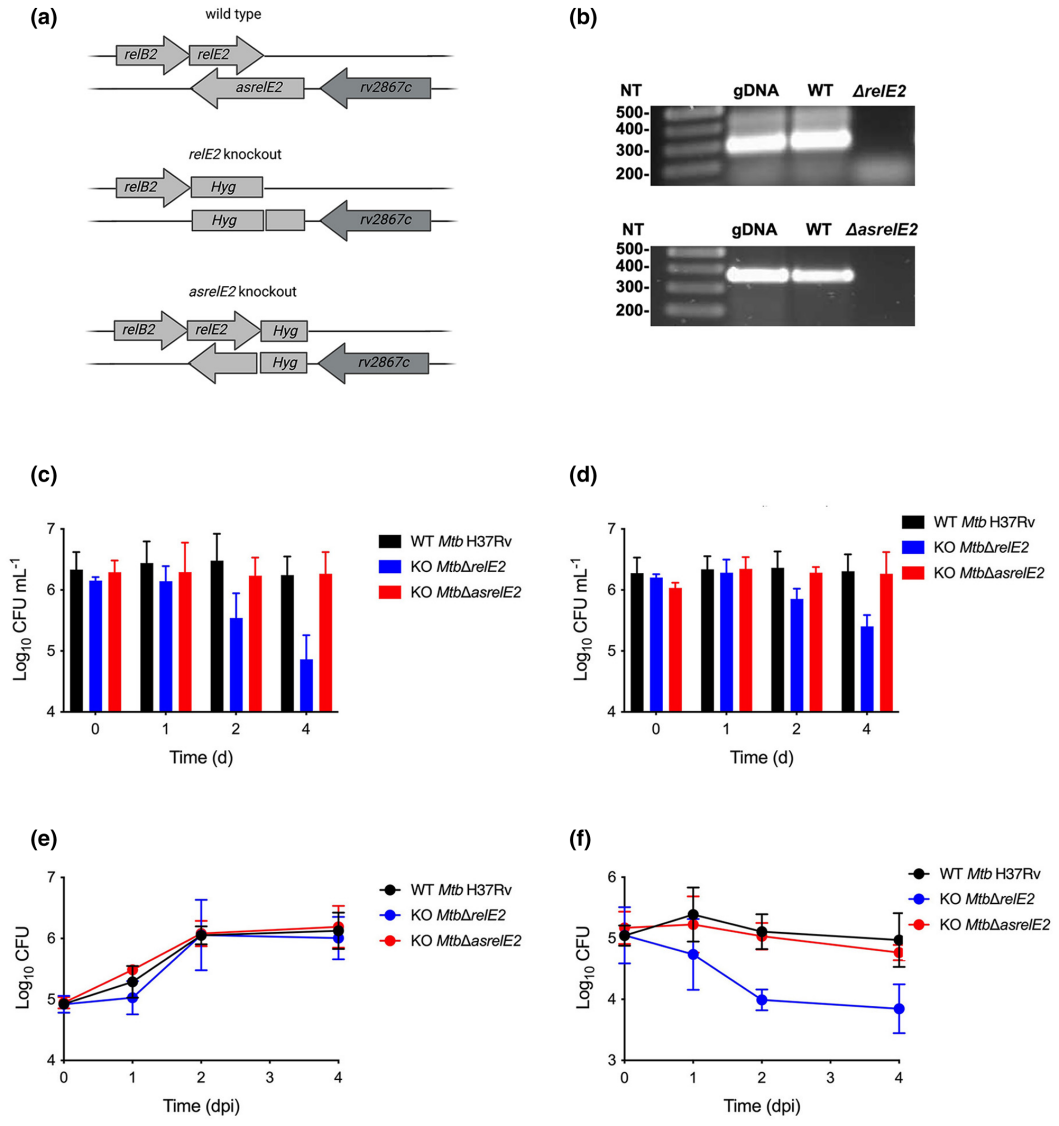
Essentiality of *relE2/asrelE2* in the survival of *Mtb* under low pH and nutrient limitation and macrophages. (a) Genetic location of RelBE2 TA loci and *relE2* and *asrelE2* knockout mutants. (b) Confirmation of *relE2* and *asrelE2* knockout mutants. Growth of WT *Mtb* and *Mtb*Δ*relE2* and *Mtb*Δ*asrelE2* mutant strains subjected to (c) pH 4.50 and nutrient limitation and (d) pH 5.50 and nutrient limitation. Growth of WT *Mtb* and *Mtb*Δ*relE2* and *Mtb*Δ*asrelE2* mutant strains in (e) resting unstimulated and (f) activated THP‐1 macrophages. Plotted data are the means ± of the standard deviations of (*N* = 3) independent experiments. Significant differences were identified using a two‐way ANOVA with Tukey's multiple comparisons post‐tests (*p‐value <0.05; **p‐value <0.01).

To discern the importance of RelE2 and asRelE2 for survival in host‐associated conditions, *Mtb*Δ*relE2* and *Mtb*Δ*asrelE2* deletion strains were constructed (Figure 6a,b) and assessed in low pH and limited nutrient conditions. Differences in growth were observed for *Mtb*Δ*relE2* and *Mtb*Δ*asrelE2* strains compared to WT *Mtb* at pH 4.5. In particular, it was observed that the *Mtb*Δ*asrelE2* strain grew slower, and the *Mtb*Δ*relE2* strain reached the stationary phase earlier than the WT control. The most significant difference between Wt *Mtb* and the mutant strains was that the survival of *Mtb*Δ*relE2* was found to steadily decrease during extended periods at pH 4.5 and NL, resulting in a nearly 20‐fold reduction compared to that of WT *Mtb* (Figure 6c). An intermediate survival phenotype was observed at pH 5.5 and NL (Figure 6d).

Macrophage ex vivo assays were also performed to assess further the role of RelB2, RelE2, and asRelE2 in survival to conditions encountered during infection. Differentiated resting and IFN‐gamma/LPS‐activated THP‐1 cells were infected with *Mtb* WT and Δ*relB2*, Δ*relE2*, or Δ*asrelE2* strains and monitored over 4 days. No significant differences in CFUs were observed in resting or activated macrophages during infection with the WT and ΔrelB2 and ΔasrelE2 knockout strains (Figure 6e,f). In contrast, a substantial decrease in CFUs was observed in activated macrophages for the Δ*relE2* knockout strain (Figure 6f). These observations substantiate that RelE2 is essential for the survival of *Mtb* under host‐associated low pH and limited nutrient stress conditions.

## 3 DISCUSSION

Adaptive responses and the bacterial NRP state involved in LTBI require alternative metabolic pathways coordinated by multiple regulatory mechanisms, including TA loci (Betts et al., 2002; Ramage et al., 2009; Ramirez et al., 2013; Salina et al., 2009; Slayden et al., 2018). We have observed that TA loci are differentially regulated in the lungs and spleen after 20 days of infection in an immune‐competent murine model of tuberculosis (Ramirez et al., 2013). The observation that cognate type II toxin and antitoxin components are differentially regulated under host‐associated stress conditions and during infection in animal models suggested the involvement of regulatory mechanisms beyond toxin‐antitoxin interactions. Accordingly, we investigated the presence of a regulatory antisense RNA, which resulted in discovering the novel asRelE2 that maps to *asrelE2* complementary to *relE2* encoded in the type II TA loci, *relBE2*. RACE studies showed that asRelE2 complements the entire *relE2* coding sequence and 248 nucleotides in the 3′ direction of the *relBE2* loci. Identifying an antisense RNA that only maps to the *relE2* portion of the *relBE2* loci is consistent with the vast regulatory antisense RNAs previously identified in *Mtb* (Arnvig et al., 2011; Arnvig & Young, 2009; Coskun et al., 2021; Schwenk & Arnvig, 2018).

The discovery of a potential regulatory antisense RNA that complements *relE2* offered the possibility that RelE2 is co‐regulated by post‐transcriptional processing at the antisense RNA level, whereby asRelE2 is involved with RNase dependent processing and alters the translation of *relE2*. Using the type I TA loci regulation mechanism of antisense RNA translational inhibition of toxins as a model, we found that the complementary portion of asRelE2 interacted with *relE2*, thus forming the required *relE2*::*asRelE2* dsRNA hybrid. We showed that the *Mtb* RNase III enzyme Rnc processed the *relE2* in an asRelE2‐dependent mechanism resulting in significantly decreased *relE2*::*asRelE2* dsRNA hybrids. The observed processing mediated by Rnc resulted in an overall reduction in *relE2*, demonstrating that the *Mtb* RNase III post‐transcriptionally regulates *relE2* in an asRelE2 dependent manner. The ability of asRelE2 to functionally regulate *relE2* was obtained from *relE2* and *asrelE2* co‐induction studies. These studies demonstrated that asRelE2 alone could rescue the observed bacteriostasis associated with RelE2. The extent of asRelE2 to regulate RelE2 was determined using our engineered recombinant fluorescence tagging system that showed co‐induction of *asrelE2* alone could comprehensively control and suppress RelE2 production. We have also verified a functional and physical interaction between RelB2 and RelE2 in live cells, thus confirming that the cognate RelB2 antitoxin could inhibit the bacteriostasis state by the toxin RelE2. These data show that asRelE2 can directly and independently control the production of the RelE2 toxin providing evidence that *relE2* is uniquely co‐regulated by an antisense mechanism and the previously defined cognate protein interactions.

There is emerging evidence that the phenotypic and adaptive diversity observed in bacterial populations is the result of multiple regulatory systems that couple bacterial cell cycle progression and metabolism with the changing growth environment (Crew et al., 2015; England et al., 2011; Ramirez et al., 2013; Schwenk & Arnvig, 2018). Many *Mtb* TA loci are induced by various adaptive responses to stress conditions (Ramage et al., 2009). This is consistent with identifying cell cycle regulators coupled with TA loci expression and adaptive responses (Crew et al., 2015; England et al., 2011; Ramirez et al., 2013). Because the regulation of *relBE2* has been associated with the stress‐responsive alternative transcription factor Crp (Kahramanoglou et al., 2014), bioinformatics searches for potential promoters and operators in proximal intergenic regions were performed. This identified a Crp binding site (CBS) in the upstream promoter region of *relBE2* as anticipated, confirming previous reports (Kahramanoglou et al., 2014). This analysis also revealed a putative convergent CBS downstream of *relBE2* on the complementary strand, indicating that Crp transcriptionally regulates *asrelE2*. We have shown that Crp, *relB2*, and *relE2* are concordantly regulated by intracellular cAMP in a pH‐dependent manner. In contrast, the transcription of *asrelE2* was slightly repressed in response to reduced pH, suggesting that asRelE2 provides proportionally greater regulation of *relE2* under conditions of increased growth. These findings establish a molecular link between the Crp regulation of RelBE2 and asRelE2 to changes in environmental pH and intracellular cAMP levels involved in regulating *Mtb* adaptive responses and virulence pathways. The demonstration that the *Mtb*Δ*relE2* mutant strain had impaired survival under low pH and nutrient‐limitation and activated macrophages substantiate that RelBE2 is necessary for survival. These data are consistent with molecular studies that have linked adaptive responses and survival of *Mtb* with increasing intracellular cAMP levels and secondary transcriptional responses (Choudhary et al., 2014; Gazdik et al., 2009; Rebollo‐Ramirez & Larrouy‐Maumus, 2019).

## 4 CONCLUSION

The survival of *Mtb* depends on its ability to adapt to changes in its environment rapidly. It is becoming increasingly clear that riboregulation is an essential co‐regulatory mechanism in adaptive responses (Schwenk & Arnvig, 2018). Our findings demonstrate that the type II TA loci *relBE2* is co‐regulated by an antisense mechanism in addition to cognate antitoxin‐interaction. The elucidation of co‐regulation by asRelE2 further highlights the importance of fine‐tuning *relBE2* in mediating the survival and persistent tolerant state in *Mtb*. This finding has significant implications regarding differential regulation of cognate TA genes and the coordination of type II TA loci in *Mtb*, and other adaptive metabolic processes necessary for infection and survival in the host throughout infection. This notion is further supported by studies linking the genome‐wide expansion of TA loci and other virulence genes to the evolution of *Mtb* (Dinan et al., 2014; Gupta et al., 2017; Sapriel & Brosch, 2019; Schwenk & Arnvig, 2018). These findings of a novel type IIb RelBE toxin‐antitoxin system in *Mtb* defined by antisense RNA co‐regulation ultimately indicate that antisense regulation of type II TA loci represents a key regulatory mechanism.

## 5 EXPERIMENTAL PROCEDURES

### 5.1 Bacteria and culture conditions

All bacterial strains used in these studies are listed in Table S1. Z‐competent *E*. *coli* strain DH5 Alpha (Zymo Research) cells were used for all cloning and propagation. One Shot® chemically competent *E. coli* strain BL21(DE3)pLysS (Invitrogen™) cells were used for the ectopic induction of recombinant *Mtb* proteins with 10 mM (final concentration) isopropyl β‐D‐1‐thiogalactopyranoside (IPTG). *E. coli* strains were grown in Luria‐Bertani (LB) broth at 37°C, shaking at 200 rpm, or on LB agar plates at 37°C. Mycobacteria were cultured in Middlebrook 7H9 broth (Difco™) supplemented with OADC (0.05 mg/ml oleic acid, 5 mg/ml BSA Fraction V, 2 mg/ml dextrose, 0.004 mg/ml catalase, and 0.85 mg/ml NaCl), 0.20% (v/v) glycerol, and 0.05% (v/v) Tween‐80 (7H9‐Tw) at 150 rpm, or on Middlebrook 7H10 agar (Difco™) plates supplemented with OADC and 0.20% glycerol at 37°C, as per standardized methods (Singh et al., 2013). For these studies, virulent *Mtb* strain H37Rv (ATCC 27294) was used as the wild‐type (WT) parental strain for constructing overexpression and mutant strains. Unless otherwise noted, optical densities at 600 nm (OD_600_) for growing *Mtb* in 7H9‐Tw of 0.10–0.30, 0.40–0.60 (or ~ 6 days), 0.70–0.90, 1.00–1.20 (or ~ 10 days), and 1.30–1.50 (or ≥ 16 days) were considered to be early‐log, mid‐log, late‐log, early‐stationary, and late‐stationary phase, respectively, as described before (Keren et al., 2011; Singh et al., 2013). Unless otherwise stated, antibiotics purchased from Sigma‐Aldrich were used at the following concentrations: 50 μg/ml ampicillin (Amp) for *E. coli*; 34 μg/ml chloramphenicol (34‐Chlor) for *E. coli*; 5 μg/ml gentamycin (Gm) for *E. coli*; hygromycin (Hyg) at 200 and 50 μg/ml for *E. coli* and *Mtb*, respectively; kanamycin (Kan) at 50 and 25 μg/ml for *E. coli* and *Mtb*, respectively.

### 5.2 Construction of *Mtb* mutant, overexpression, and reporter strains

The *Mtb* Δ*relE2* and Δ*asrelE2* deletion mutant strains were generated using a two‐step allelic exchange with a temperature‐sensitive replicative vector, pPR27‐*xylE*, as initially described by (Pelicic et al., 1997). In brief, to create mutant strains, ~1000 base‐pairs (bp) upstream and downstream flanking the 264‐bp *relE2* gene and the 372‐bp intergenic region (IGR) intervening in between *relE2* and *rv2867c* were PCR amplified from *Mtb* str. H37Rv genomic (g)DNA with gene‐specific primers (GSPs) in Table S2 using GoTaq® Green (2X) Master Mix (Promega Corporation) enriched with 5% (final concentration) PCR‐grade DMSO (Sigma‐Aldrich) per the manufacturer's notes (Promega Corporation). For the generation of *Mtb*Δ*relE2*, the upstream and downstream regions were cloned into a mycobacterial shuttle vector, pVV16, flanking the hygromycin resistance (Hyg^R^) gene *hph*. The resulting 4049‐bp dsDNA fragment, *rv2864c‐relB2*‐*hph*‐*rv2867c*, was excised and then cloned into pPR27‐*xylE* at NotI and SpeI restriction digest (RD) sites for sucrose (Suc) counter selection. To develop *Mtb*Δ*asrelE2*, upstream and downstream regions were cloned into a mycobacterial shuttle vector, pMIND, flanking the Kan‐resistance (Kan^R^) gene *aphA*. The resulting 3800 bp fragment, *rv2864c*‐*relBE2*‐*aphA*‐*rv2867c*, was then cloned into pPR27‐*xylE* at NotI and XbaI RD sites, creating pPR27‐*asrelE2*KO. Freshly prepared electrocompetent WT *Mtb* H37Rv was electroporated with various allelic exchange vectors. Following the outgrowth of Hyg^R^ and Suc^R^ colonies in 7H9‐Tw with 50‐Hyg (*Mtb*Δ*relE2*) and Kan^R^ and Suc^R^ in 7H9‐Tw with 25‐Kan (*Mtb*Δas*relE2*) for 4 weeks at 37°C, successful deletions from genomes of mutant *Mtb* strains were confirmed by PCR analyses using GSPs listed in Table S2.

For overexpression studies in *Mtb*, *relE* toxins were PCR amplified from gDNA using forward GSPs, producing N‐terminal tetra‐cysteine tags for in situ protein detection. Controlled overexpression was achieved using anhydrotetracycline (ATc)‐inducible overexpression vector, pST‐KT, essentially as first reported by (Parikh et al., 2013). RelE2^ΔR61L^ and RelE2^ΔR81L,Y85F^ toxin genes were constructed by changing G‐182 and G‐242 and A‐254 to T based on prior reporting (Neubauer et al., 2009), using reverse GSPs with single nucleotide polymorphisms (SNPs) in PCRs Table S2. For co‐overexpression of antitoxin genes, P_myc1_ *tetO1* was PCR amplified from pST‐KT and re‐cloned into ATc‐inducible pE2 derivatives, thereby creating a duplicate promoter P_myc2_ *tetO2*. *Mtb* RelB2 and asRelE2 antitoxin genes were cloned in NotI, and HindIII RD sites were engineered immediately downstream of P_myc2_ *tetO2*. *Mtb* was electrotransformed with *relBE2* and *asrelE2* overexpression vectors listed in Table S1, as reported before (Parish & Stoker, 1998), and incubated at 37°C on 7H10 agar with 25‐Kan for 3–4 weeks or until colonies became visible.

For dual transcriptional reporter assays, pGREENCHERRY plasmids were constructed, encoding the pH‐sensitive green fluorescent protein (GFP) (Vandal et al., 2008) regulated by the *Mtb relBE2* promoter, containing a Crp binding site (CBS), P_*relBE2*_CBS, and mCHERRY (Carroll et al., 2010), which is controlled by the convergent *Mtb asrelE2* promoter, containing another CBS, P_*asrelE2*_CBS. Initially, a constitutive promoter P_smyc_ was excised from pCHERRY3 and replaced with the ~120‐bp P_*asrelE2*_CBS intervening between *asrelE2* and *rv2867c*. The mCHERRY gene was PCR amplified using GSPs in Table S2 and re‐cloned into pCHERRY3 to add a C‐terminal tag (ADSHQRDYALAA) encoded by SsrA (MTB000042). This fusion tag enhances the mCHERRY decay (Andersen et al., 1998; Personne & Parish, 2014). The 105‐bp P_*relBE2*_CBS encoded between *rv2864c* and *relBE2* was subsequently cloned into the pCHERRY derivative. Then, the GFP gene from pUV15‐*pHGFP* made available by (Vandal et al., 2008), was PCR amplified, producing an additional fusion C‐terminal SsrA decay tag, and re‐cloned downstream of P_*relBE2*_CBS at ClaI and SpeI RD sites, creating a WT dual transcriptional reporter pGREENCHERRY^WT^ (Table S1). For the construction of nonfunctional mutant dual transcriptional reporter, pGREENCHERRY^Mut^, P_*relBE2*_CBS and P_*asrelE2*_CBS were PCR amplified using forward GSPs, making their CBSs non‐functional. Specifically, the left arm of P_*relBE2*_CBS (tGAGa) was mutated to tGGAa, while the left arm of P_*asrelE2*_CBS (cGACg) was mutated to cGCAg, ablating Crp‐DNA‐binding, as shown before (Agarwal et al., 2006; Rickman et al., 2005). Mut P_*asrelE2*_CBS and P_*relBE2*_CBS PCR amplicons were then cloned into pGREENCHERRY similarly, and transcriptional reporters were electroporated into WT *Mtb* H37Rv.

### 5.3 Functional interaction analysis of *Mtb* relBE2/asrelE2 TA genes

*Mtb relBE2/asrelE2* merodiploid strains in Table S2 were incubated shaking at 150 rpm and 37°C for at least 16 days to the late‐stationary phase. These cultures were then diluted in 150 ml fresh 7H9‐Tw with 25‐Kan to an OD_600_ of ~0.10, and 150 μl of 2 mg/ml ATc (Takara™) was added to induce the expression of *relBE2/asrelE2* TA genes. Ectopic inductions were carried out at 37°C and 150 rpm for up to 20 days in the dark, and for every 2 days, OD_600_, CFU/ml, and N‐tetracysteine‐RelE2 fluorescence (RFU or excitation/emission = 508 nm/528 nm) were assessed. To measure in situ RFUs, up to 10 ml of ectopically induced cultures were washed three times in TBST (pH 6.50), resuspended in 500 μl of 10% formalin (Sigma‐Aldrich), containing 20 μM FlAsH‐EDT2 biarsenical labeling reagent (Invitrogen™), and fixed in the dark at 4°C for 2 d. Formalin‐fixed tubercle bacilli were rinsed twice in BAL wash buffer per the manufacturer's instructions (Invitrogen™) and resuspended in TBST (pH 6.50). The whole‐cell RFUs were measured with an EnSpire Multimode microplate reader (PerkinElmer) and normalized to OD_600_.

### 5.4 Physical interaction analysis of *Mtb* RelBE2 TA proteins

RelBE2 TA protein–protein interaction studies were performed as described by (Ramirez et al., 2013), with few modifications. In brief, RelB2 and RelE2 gene fragments were amplified from *Mtb* H37Rv gDNA using GoTaq® Green (2X) Master Mix (Promega Corporation) enriched with 5% PCR‐grade DMSO (Sigma‐Aldrich) per the manufacturer's notes (Promega Corporation), and cloned into pET28a and pETcoco2, respectively (Table S1). DNA constructs were transformed into *E. coli* strain DH5 Alpha (Zymo Research) and transformants were selected from overnight growth at 37°C on LB agar with 50‐Kan for pET28a and 50‐Amp for pETcoco2. Sequenced vectors were transformed into chemically competent *E. coli* strain BL21(DE3)pLysS (Invitrogen™) cells. The selection was carried out overnight by growth in LB broth supplemented with 34‐Chlor 50‐Kan for pET28‐*relB2* selection or 50‐Amp for pETcoco2‐*relE2* selection, or both for co‐transformation. Overnight cultures were then diluted 1–50 into fresh LB media containing the necessary antibiotics. When the pETcoco2‐*relB2* construct was used, LB media had 0.01% (v:v) L‐arabinose to amplify plasmid copy number before ectopic induction. Once subcultures reached an OD_600_ of ~0.50, protein production was induced by adding 10 mM (final concentration) IPTG. Subcultures were incubated for another 5 h at 150 rpm and 37°C, and bacterial cell pellets were collected via brief centrifugation. According to the manufacturer's protocols, crude whole cell lysates were obtained using BugBuster® with Benzoase® (Novagen). Crude whole cell lysates were then clarified by centrifugation at 12,500 × *g* for 20 min at 4°C and passed through a 0.20 μM filter. Each mL of clarified lysate was combined with 250 μl of pre‐washed Ni‐NTA His‐Bind® Resin (Qiagen) and rocked gently at 4°C for about 1 hour before packing into a column with 10 ml of bind buffer (100 mM Tris–HCl, 250 mM NaCl, and 5 mM imidazole, pH 7.80). The column was rinsed three times with wash buffer‐one (100 mM Tris–HCl and 250 mM NaCl, 10 mM imidazole, pH 7.80), and then three times with wash buffer‐two (100 mM Tris–HCl and 250 mM NaCl, 25 mM imidazole, pH 7.80). Recombinant TA proteins and/or protein complexes were eluted stepwise in elution buffer (100 mM Tris–HCl, 500 mM NaCl, pH 7.80) containing 50‐, 125‐, and 250‐mM imidazole. All wash and elution fractions were separated on NuPAGE® 12% Bis‐Tris Gels (Invitrogen™) in MES running buffer (Invitrogen™) at 200 V, followed by transfer to a 0.2micron nitrocellulose membrane (BioRad) at 50 V for western blotting. Membranes were blocked in 4% BSA in TBST (pH 7.60), incubated with primary Penta‐His antibody (Qiagen) or anti‐HSV‐Tag® antibody (Novagen), diluted at 1:10,000, followed by goat anti‐mouse‐alkaline phosphatase (Sigma Aldrich), diluted 1:10,000. Membranes were developed with the addition of NBT/BCIP substrate solution (Sigma‐Aldrich).

### 5.5 Extraction and purification of *Mtb* total RNA

Total RNA was isolated from 50 ml culture aliquots of *Mtb*. Bacilli were collected by centrifugation at 3500 × *g* for 10 min at 4°C, washed two times in TBST (pH 6.50), and resuspended in 1 ml of TRIzol® Reagent (Invitrogen™). Bacilli were lysed by physical disruption in 1.50 ml screw‐cap tubes (USA Scientific) with 250 μl of 0.10 mm zirconia glass beads (BioSpec Products) subjected to 2400 oscillations for 30 seconds six times, using the Mini‐BeadBeater‐1 (BioSpec Products), with cooling on ice for 2 min in between each round of bead beating. Following the disruption, 200 μl of chloroform was mixed by vigorous vortexing for 15 seconds, and whole‐cell lysates were centrifuged at 12,500 × *g* for 15 min at 4°C. 500 μl of the aqueous layers were transferred to new 1.50 ml microcentrifuge tubes containing 500 μl of ice‐cold molecular biology grade isopropanol (Sigma‐Aldrich), vortexed, incubated at ‐20°C overnight, and centrifuged at 12,500 × *g* for 15 min at 4°C to pellet RNA. RNA pellets were washed once in 80% molecular biology grade 200‐proof ethanol (Sigma‐Aldrich) in DEPC‐treated H_2_O (Sigma‐Aldrich), dried at room temperature, and treated with 10 units (U) DNase I (Thermo Scientific™) at 37°C for 60 min. Equal volumes of phenol:chloroform (5:1) pH 4.30–4.70 (Sigma‐Aldrich) were mixed with DNase I reactions with vigorous vortexing for 15 seconds and centrifuged at 12,500 × *g* for 3 min at 4°C. Top aqueous layers were transferred to new 1.5 ml microcentrifuge tubes with 10 volumes of 80% ethanol, 10% 3 M sodium acetate (Sigma‐Aldrich), and 0.50 μg/ml glycogen (ThermoFisher Scientific™) in DEPC‐treated H_2_O and incubated at ‐20°C overnight to precipitate RNA. Following three rounds of DNase I treatment, total RNA was quantified and qualified using the NanoDrop (ND‐1000) UV/VIS Spectrophotometer (ThermoFisher Scientific™), and only samples with absorbance ratios at 260–280 nm of 1.90–2.00 were used in downstream gene expression analyses.

### 5.6 Northern blotting analysis of *Mtb* total RNA

Northern blotting of *Mtb* total RNA was performed as reported before (Gerrick et al., 2018), using 5′‐ and 3′‐digoxigenin (DIG)‐labeled riboprobes listed in Table S2, which were synthesized by IDT DNA Technologies (Coralville, IA). Approximately 5 μg of total RNA samples were heated to 75°C for 5–10 min in (2X) TBE‐urea sample buffer (Invitrogen™), run on 6% TBE‐urea gels in (1X) TBE buffer (Invitrogen™) at 180 V for 45 min, transferred to Ambion® BrightStar® positively charged nylon membranes at 30 V for 60 min using the XCell II™ Blot Module (Thermo Fisher Scientific), and crosslinked using the UV Stratalinker® 1800 per the manufacturers' notes (Stratagene). UV crosslinked transferred membranes were prehybridized in ULTRAhyb™ Ultrasensitive Hybridization Buffer (Thermo Fisher Scientific) for 60 min at 68°C before adding riboprobes and then incubated overnight at 68°C with gentle movement. Membranes were washed twice with (0.5X) SSC NorthernMax™ Low Stringency Wash Buffer (Invitrogen™) at 68°C. Membranes were washed, rinsed, and blocked for 30 min at room temperature with (1X) DIG Wash and Block Buffers (Roche), respectively, and then incubated with 1:2500 (final concentration) anti‐DIG‐AP‐conjugate in (1X) DIG block buffer. Northern blots were developed using the DIG Nucleic Acid Detection Kit per the manufacturer's protocol (Roche) and imaged using ChemiDoc™ XRS^+^ (Bio‐Rad).

### 5.7 5′/3′ rapid amplification of complementary ends (RACE) of *Mtb* total RNA

5′/3′ RACE was applied as reported before (Schifano et al., 2014) to three pools of RNA: 5′ 3PO_4_ primary RNA; 5′ PO_4_ processed RNA; 5′ OH cleaved RNA. For primary transcripts, around 2 μg of total RNA was incubated along with 2 U of 5′ PO_4_‐dependent riboexonuclease (Lucigen) for 60 min at 30°C to selectively degrade 5′ PO_4_ RNAs, followed by 100 U RNA pyrophosphhydrolase (RppH–NEB) to remove pyrophosphate from 5′ 3PO_4_ ends, and then with 10 U of T4 RNA Ligase I (ThermoFisher Scientific) and 0.10 mg/ml BSA for 3 h at 37°C and overnight at 16°C to attach the 5′ RNA oligo adaptor (Table S2) to 5′ PO_4_ ends. To select 5′ PO_4_ processed RNA, equal amounts (μg) of total RNA and RNA adaptor were similarly incubated with T4 RNA ligase I and BSA. For the selection of 5′ OH RNAs, 2 μg of total RNA was treated with 4 U of RppH, 2 U of 5′ PO_4_‐dependent riboexonuclease (Lucigen), 2 U of T4 polynucleotide kinase (ThermoFisher Scientific) at 37°C for 30 min to phosphorylate 5′ OH ends, and then T4 RNA ligase I and BSA. After extracting RNA in acid‐phenol:chloroform and precipitating overnight in 0.50 μg/ml glycogen at ‐20°C, 2 μg of 5′ ligated RNA pools were incubated with 2 U of *E*. *coli* poly(A) polymerase (NEB) at 37°C for 30 min to polyadenylate 3′ ends and reverse transcribed with Oligo(dT)_20_ using the Transcriptor First Strand cDNA Synthesis Kit (Roche) at 50°C for 60 min. Single‐stranded cDNA was column purified (Zymo Research) and PCR‐amplified using 0.40 μM forward adaptor‐specific primer and reverse GSPs in Table S2 in GoTaq® Green Master Mix (Promega) and 0.16 mg/ml PCR grade DMSO added for 5′ RACE. 3′ RACE was performed essentially the same but with nested forward GSPs and a reverse Oligo(dT)_20_‐specific primer listed in Table S2. 5′/3′ RACE PCR products were run on 1.5% agarose gels at 95 V for ~70 min in (1X) TAE, gel purified, cloned into pMIND, and Sanger sequenced.

### 5.8 Double‐stranded (ds)RNA cleavage assay with *Mtb* RNase III

N‐terminal hexahistidine tagged *Mtb* RNase III (His‐RNase III) was overproduced and purified essentially as reported before (Akey & Berger, 2005). *Mtb* H37Rv *RNase III* was PCR amplified from gDNA, cloned into pETcoco2, and overproduced in *E*. *coli* BL21(DE3)pLysS (Invitrogen™) at an OD_600_ of ~0.50 and 37°C with 1 mM of IPTG for 5 h. Harvested cells were resuspended in BugBuster™ Reagent (Millipore) with EDTA‐free protease inhibitor (Roche) and 250 U of Benzoase Nuclease (Novagen), lysed at room temperature, rocking gently for 30 min, and centrifuged at 12,500 × *g* for 20 min at 4°C. The clarified whole cell lysate was incubated with Ni‐NTA His•Bind® resin (Millipore) for 60 min at 4°C, rocking gently, and loaded onto a column pre‐equilibrated with ice‐cold buffer (Tris–HCl [pH 7.90] and 500 mM NaCl) with 10 mM imidazole. The column was washed with six volumes of ice‐cold buffer with 50 mM imidazole. His‐RNase III was eluted in three volumes of ice‐cold buffer with 250 mM imidazole. Elution fractions were pooled into a 3 kDa MWCO Amicon Ultra‐15 Centrifugal Filter Unit (Millipore) and dialyzed against at 4°C in Tris–HCl (pH 7.90) and 5% glycerol with 500, 250, and 150 mM NaCl. His‐RNase III was resolved on 12% Bis‐Tris gels (Invitrogen™), stained with SimplyBlue SafeStain (ThermoFisher Scientific), estimated to be at least 80% pure quantified using BCA assay (ThermoFisher Scientific), and stored at ‐20°C until further use.

In vitro, His‐RNase III dsRNA cleavage assays were performed as recently published by Gordon et al. (2017). Full‐length *relE2* and *asrelE2‐3* DNA templates were PCR amplified from *Mtb* H37Rv gDNA using GoTaq® Green Master Mix (Promega) with GSPs adding 5′‐TAATACGACTCACTATAGGG‐3′ upstream of T7 promoters (Table S2), and gel purified. RNA was in vitro transcribed using T7 RiboMAX Express large‐scale RNA Production System (Promega) and then purified by acid‐phenol: chloroform (pH 4.50) with overnight ethanol precipitation at −20°C. Approximately 400.00 ng/μl (final concentration) of in vitro transcribed RNA was mixed with DEPC‐treated H_2_O and (5X) dsRNA cleavage buffer (150 mM Tris–HCl (pH 7.60), 250 mM NaCl, 0.50 mM EDTA, and 0.50 mM DTT) to create 50 μl reactions, heated to 70°C for 10 min, and immediately cooled on ice. One μg of His‐RNase III and 5 μl of 0.10–50.00 mM MgCl_2_ were added on ice. RNase III dsRNA cleavage reactions, including negative control reactions with one μg of His‐RNase III, 50 mM MgCl_2_, and 1 μg of *relE2* or asRelE2‐3, were incubated at 37°C for 30 min, quenched with the addition of 5 μl of 440 mM EDTA, and RNA was extracted with acid‐phenol‐chloroform and precipitated overnight as described above. Five microliters of 1 μg/μl RNA isolated from RNase III dsRNA cleavage reactions were mixed with (2X) TBE‐urea sample buffer (Invitrogen™), heated to 75°C for 5 min, centrifuged at 6000 × *g* for 3 min at 4°C, and separated on 6% TBE‐urea gels in (1X) TBE buffer (Invitrogen™) at 180 V for 50 min. Resolved gels were stained in SYBR® Gold (Invitrogen™) for 45 min and imaged using ChemiDoc™ XRS^+^ (Bio‐Rad).

### 5.9 *Mtb* in vitro stress and persistence assays

Mid‐to‐late stationary phase cultures of WT and mutant *Mtb* strains were diluted to an OD_600_ of ~0.10 in 75 ml of 7H9‐Tw with 50‐Hyg or 25‐Kan, and subcultured at 37°C and 150 rpm for 20 days. During this time, outgrowth was assessed by measuring OD_600_ and enumerating CFU/ml from plating 10‐fold serial dilutions of culture aliquots onto 7H10 agar with antibiotics every 2 days. Results represent the means ± of the standard deviation of at least three independent experiments. To assess the effects of in vitro stress conditions associated with the host, WT and mutant *Mtb* cultures were treated similarly as reported before (Betts et al., 2002; Early et al., 2019; Singh et al., 2013). In brief, *Mtb* strains were grown in 7H9‐Tw with 50‐Hyg or 25‐Kan until the mid‐to‐late log phase. Three 50 ml culture sample aliquots were briefly centrifuged at ~4500 × *g* for 10 min at 4°C, washed twice, and then resuspended to an OD_600_ of 0.20–0.25 (or 6.50‐to‐7.00 Log_10_ CFU/ml) in (1X) TBST (i.e., 20 mM Tris, 150 mM NaCl, and 0.05% nonmetabolizable tyloxapol). For cAMP studies, 50 ml cell aliquots washed and resuspended in TBST pH 6.50 were incubated with 0–10 mM (final concentration) of whole‐cell soluble analog dibutyryl cyclic adenosine monophosphate (db‐cAMP), rocking gently, at 37°C for 2 days, as published before (Agarwal et al., 2006). At 0, 1, 4, 24, 48 h post‐exposure to db‐cAMP, bacilli were centrifuged at 4500 × *g* for 10 min at 4°C, washed two times, and resuspended in TBST (pH 6.50). For assessing intracellular cAMP levels, 1 ml sample aliquots were boiled in 0.1 M HCl and then stored at ‐80°C until assayed. For measuring whole‐cell fluorescence, up to 10 ml of sample aliquots were fixed in 10% formalin (Sigma‐Aldrich) for 2 days at 4°C before reading GFP RFUs (excitation/emission = 395 nm/510 nm) and mCHERRY RFUs (excitation/emission = 587 nm/610 nm) using the EnSpire Multimode microplate reader (PerkinElmer). Corresponding 1 ml sample aliquots were serially diluted and plated on 7H10 agar with 50‐Hyg or 25‐Kan when necessary for enumerating Log_10_ CFUs. For low pH stress, 50 ml sample aliquots were washed twice and resuspended in TBST at a pH of 6.50, 5.50, or 4.50 to an OD_600_ ~ 0.25 (or 6.50–7.00 Log_10_ CFU/ml) and incubated, rocking gently for 8 days at 37°C. Following 0, 1, 2, 4, and 8 days of acid stress, bacilli were enumerated as described above: (1) 1 ml was prepped to estimate intracellular cAMP levels; (2) up to 10 ml was fixed in 10% neutral buffered formalin to assess GFP and mCHERRY RFUs; (3) 1 ml was serially diluted and plated onto 7H10 agar with antibiotics when necessary to determine Log_10_ CFUs. Significant differences in survival of various WT and mutant strains were made by comparing means ± of the standard deviations of three independent experiments using a two‐way ANOVA with Tukey's post‐tests (*p < 0.05, **p < 0.01, and ***p < 0.001), as recently reported (Gallant et al., 2016).

### 5.10 Measurement of intracellular *Mtb* cAMP levels

Intracellular *Mtb* cAMP levels were measured using the Direct cAMP Enzyme Immunoassay Kit according to the acetylated version of the manufacturer's protocol (Sigma‐Aldrich). Sample culture aliquots were recovered and resuspended to ~1x10^8^ CFU/ml in TBST, pH 6.50, centrifuged at 4500 × *g* for 10 min at 4°C, resuspended in 0.10 M HCl, and boiled for 10 min at 100°C (Kahramanoglou et al., 2014). Whole‐cell lysates were transferred to 1.50 ml screw‐cap microcentrifuge tubes (USA Scientific) filled with 200 μl 0.10 mm diameter zirconia glass beads (BioSpec Products) and exposed to three rounds of bead beating (2400 oscillations in 30 s), using the Mini‐BeadBeater‐1 (BioSpec Products), with cooling on ice for at least 2 min in between each round. Bacterial cell debris was removed via centrifugation at 12,500 × *g* for 15 min at 4°C, and clarified lysates were stored at −20°C until further use. Intracellular cAMP levels were measured by reading the optical density at 405 nm (OD_405_) of 100 μl of immunoassay whole cell lysates using an EnSpire Multimode microplate reader (PerkinElmer). Intracellular cAMP levels were estimated from standard curves generated from reading the OD_405_ of 0–20 pmol/ml of cAMP in 0.10 M HCl, and cAMP per 10^8^ CFU was calculated by dividing pmol cAMP/ml by CFU/ml, similarly to prior reporting (VanderVen et al., 2015).

### 5.11 Reverse transcription‐quantitative PCR (RT‐qPCR) of *Mtb* and murine total RNA

For RT‐qPCR gene expression analyses, 1 μg of total RNA was heated to 65°C for 10 min with 2.50 μM reverse GSPs, cooled to 4°C, and mixed with 20 U of transcriptor RT reverse transcriptase and 80 U of RNase inhibitor and reverse transcribed at 58°C for 60 min. No RT (NRTs) and no template controls (NTCs) were included with every reaction. Four microliters of 1:25 and 1:50 dilutions of cDNA were used in 25.00 μl qPCR reactions containing 12.50 μl (2X) SYBR Green I Master Mix (Roche), 2 μl of 5 μM of forward and reverse GSPs (Table S2), 2 μl of DMSO, and 4.50 μl of DEPC‐treated H_2_O carried out on the LightCycler® 480 System per the manufacturer's instructions (Roche). GSPs were optimized by generating standard curves of qPCRs of cDNA (or Cp values) reverse transcribed from serially diluted early‐to‐mid‐log phase total RNA (0 h). Amplification efficiencies (Es) were determined using linear regression analyses (E = 10^−1/slope^), and GSPs with at least 85% qPCR E was used for relative quantification (Figure S1). Genes of interest (GOIs) were normalized to 16S rRNA (MTB000019), and fold inductions were calculated using E ^Δ*GOI*^/E ^Δ*16S*^ for *Mtb* and E^Δ*GOI*^/E^Δ*β*−*Actin*^ in comparison to 0 controls (Livak & Schmittgen, 2001). Melt curve analyses were run in tandem to confirm qPCR amplicon specificity, and mean fold inductions ± standard deviations were calculated from at least three independent experiments.

### 5.12 *Mtb* infection of THP‐1 cells

Human monocytic THP‐1 cells (ATCC TIB‐202) were maintained in RPMI‐1640 (ATCC 30–2001) culture medium supplemented with 10% fetal bovine serum (FBS, ATCC 30–2020) and 0.05 mM beta‐mercaptoethanol (Sigma) at 37°C, 5% CO2. THP‐1 cells were seeded in flat‐bottom 24‐well plates at 5 × 10^5^ cells/well and treated overnight with 100 nM phorbol 12‐myristate 13‐acetate (PMA, Sigma). The resulting differentiated cells were incubated in supplemented RPMI without PMA for 24 h. Activated macrophages were established by incubating differentiated cells with 20 ng/ml IFN‐gamma (R&D Systems) and 20 pg/ml LPS (Sigma) for 16 h. Both activated, and nonactivated macrophages were infected with log‐phase *Mtb* H37Rv WT and KO cultures at an MOI of 10 for 4 h. The remaining inoculum was serially diluted and plated in duplicate on 7H11 agar plates for CFU enumeration. Cells were washed twice with sterile PBS following the incubation period to remove extracellular bacilli. At each desired time post‐infection, infected cells were lysed with 0.05% SDS in 7H9 broth. Replicate cell lysates were pooled and centrifuged at 3500 × g for 10 min to pellet intracellular bacteria. Pellets were resuspended in 7H9 broth, serially diluted, and plated in duplicate for CFU determination.

## CONFLICT OF INTEREST

The authors have no conflict of interest to declare.

## AUTHOR CONTRIBUTIONS

CCD and RAS concieved original project design. CCD, JEC and JMS performed experimentation. CCD, JEC, JMS and RAS performed data analysis. All authors contributed to original draft, CCD and RAS were responsible for final draft. RAS obtained funding for this research, provided resources and project supervision. All authors contributed to the article and approved the submitted version.

## Supporting information


Figure S1

Tables

## Data Availability

The data that support the findings of this study are available from the corresponding author upon reasonable request.
