# Supplementary figures and images for: Discovery of a novel type IIb RelBE toxin‐antitoxin system in Mycobacterium tuberculosis defined by co‐regulation with an antisense RNA
Source: Mol Microbiol. 2022 May 24;117(6):1419–33. doi: 10.1111/mmi.14917 (PMC9325379; doi:10.1111/mmi.14917)

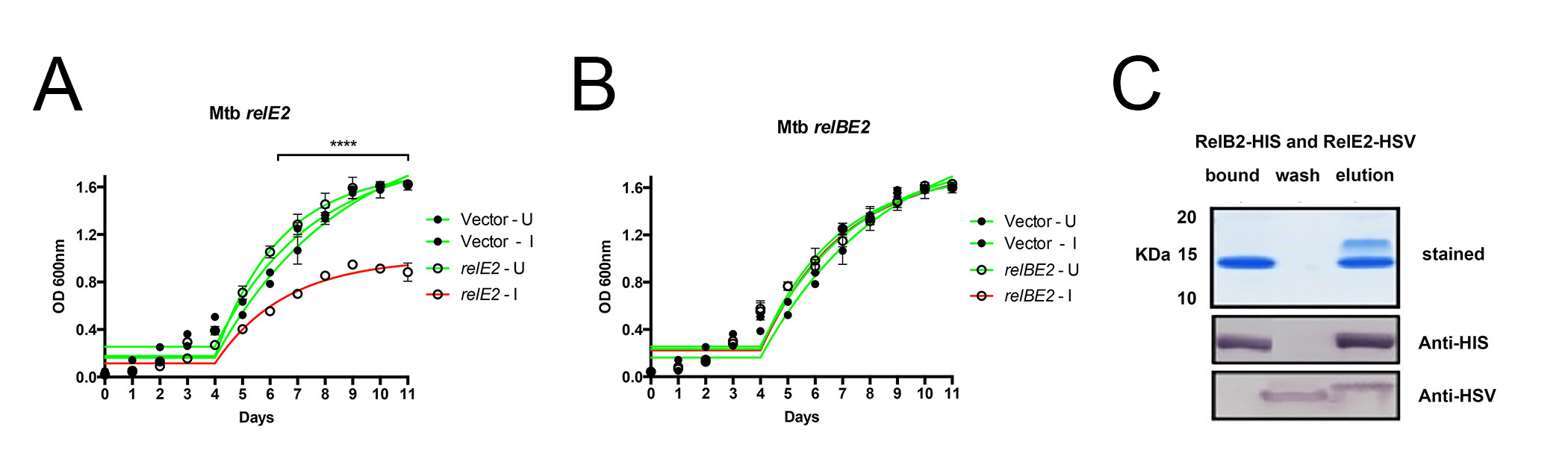

Supplement: Supplementary file 1 — Figure S1 [file MMI-117-1419-s001.tif]
