# Supplementary material for: Discovery of a novel type IIb RelBE toxin‐antitoxin system in Mycobacterium tuberculosis defined by co‐regulation with an antisense RNA
Source: Mol Microbiol. 2022 May 24;117(6):1419–33. doi: 10.1111/mmi.14917 (PMC9325379; doi:10.1111/mmi.14917)
Supplement: Supplementary file 2 — Tables [file MMI-117-1419-s002.docx]

**Supplemental Table 1: Strains and plasmids used in this study.**

| Strains and Plasmids | Additional Information | Source and Reference |
| --- | --- | --- |
| Strains |  |  |
| *E. coli* strain DH5α | F^–^ φ80*lacZ*∆M15 *∆*(*lacZYA-arg*F)U169 *deoR*, recA1 *end*A1 *hsd*R17(rK^–^, mK^+^) *pho*A *sup*E44 *λ*^–^ *thi*-1 *gyr*A96 *rel*A1 | Zymo Research |
| *E. coli* strain BL21(DE3)pLysS | F^–^ *omp*T *hsd*S_B_ (r_B_^–^, s_B_^–^) *gal dcm* (DE3) pLysS(Cam^R^) | Invitrogen^TM^ |
| *Mtb* strain H37Rv | Virulent *Mtb* strain ATCC ® 27294^TM^ | ([Steenken *et al.*, 1934](#_ENREF_70)) |
| *Mtb*Δ*relE2* | *Mtb* H37Rv *relE2* mutant strain with Hyg^R^ | This study |
| *Mtb*Δ*asrelE2* | *Mtb* H37Rv *asrelE2* mutant strain with Kan^R^ | This study |
| Plasmids |  |  |
| pVV16 | Constitutive overexpression mycobacterial shuttle vector encoding Hyg^R^ | Kindly provided by Karen Dobos (TBVRM, Colorado State University) |
| pMIND | Tetracycline-inducible overexpression mycobacterial shuttle vector encoding Kan^R^ | Made available by Brian Robertson at Addgene; ([Blokpoel *et al.*, 2005](#_ENREF_11)) |
| pPR27-*xylE* | Thermosensitive replicative *Mtb* vector with sucrose counterselection and Gm^R^ | Kindly provided by Mary Jackson ([Pelicic *et al.*, 1997](#_ENREF_51)) |
| pPR27-*relE2*KO | pPR27-*xylE* with pVV16 Hyg^R^ gene *hph* flanked by *rv2864c*-*relB2* and *rv2867c* | This study |
| pPR27-*asrelE2*KO | pPR27-*xylE* with pMIND Kan^R^ gene *aphA* flanked by *rv2864c*-*relBE2* and *rv2867c* | This study |
| pST-KT | Mycobacterial non-toxic ATc-inducible overexpression vector encoding Kan^R^ | Made available by Vinay Nandicoori at Addgene; ([Parikh *et al.*, 2013](#_ENREF_48)) |
| pE2^WT^ | pST-KT encoding ATc-inducible RelE2^WT^ | This study |
| pE2^ΔR61L^ | pST-KT encoding ATc-inducible RelE2^ΔR61L^ | This study |
| pE2^ΔR81L,Y85F^ | pST-KT encoding ATc-inducible RelE2^ΔR81L,Y85F^ | This study |
| pBE2^WT^ | pE2^WT^ encoding RelB2^WT^ controlled by P_myc2_*tetO2* | This study |
| pASE2^WT^ | pERRY2^WT^ encoding asRelE2^WT^ controlled by P_myc2_*tetO2* | This study |
| pET28a | N- and C-terminal His_6_ tag IPTG-inducible overexpression vector with Kan^R^ | Novagen |
| pET28a-*his-relB2-his* | pET28a encoding IPTG inducible N- and C-terminal His^6^-tagged Mtb RelB2 and Kan^R^ | This study |
| pETcoco2 | N-terminal His_6_ tag and C-terminal HSV tag IPTG-inducible overexpression vector with Amp^R^ | Novagen |
| pETcoco2-*relE2-hsv* | pETcoco2 encoding IPTG-inducible C-terminal HSV-tagged *Mtb* RelE2 and Amp^R^ | This study |
| pETcoco2-*his-rnaseIII* | pETcoco2 encoding IPTG-inducible N-terminal His_6_-tagged *Mtb* RNase III and Amp^R^ | This study |
| pETcoco2-*his-crp* | pETcoco2 encoding IPTG-inducible N-terminal His_6_-tagged *Mtb* Crp and Amp^R^ | This study |
| pCHERRY3 | *Mtb* reporter plasmid encoding codon-optimized mCHERRY and Hyg^R^ | Made available by Tanya Parish at Addgene; ([Carroll *et al.*, 2010](#_ENREF_13)) |
| pUV15-*pHGFP* | *Mtb* reporter plasmid encoding codon-optimized pH-sensitive GFP and Hyg^R^ | Made available by Sabine Ehrt at Addgene; ([Vandal *et al.*, 2008](#_ENREF_77)) |
| pGREENCHERRY^WT^ | pCHERRY3 encoding mCHERRY-SsrA and pHGFP-SsrA controlled by WT P*_asrelE2_*CBS and *P_relBE2_*CBS | This study |
| pGREENCHERRY^Mut^ | pCHERRY3 encoding mCHERRY and GFP controlled by Mut P*_asrelE2_*CBS and P*_relBE2_*CBS | This study |

**Supplemental Table 2: Oligonucleotides used in this study.**

| Oligo function and gene(s) or region of interest | Forward Primer (5’-to-3’) | Reverse Primer (5’-to-3’) |
| --- | --- | --- |
| Allelic Exchange | | |
| *relE2*-up | CCCAAAGCGGCCGCCGGTGTGATCACCACGCC | TTTGGGGGTACCTCAGTGGGGGCGTCGCG |
| *relE2*-down | CCCAAATCTAGAAACTCACCGACGGGCGCT | TTTGGGCATATGTTACTAGTTGTACGGGGCGAAGTGATC |
| *relE2*-KO | GTGCCTTACACCGTGCGG | CTATCGGCGGTAGATGTCC |
| *asRelE2*-up | CCCAAACATATGGCGGCCGCCGGTGTGATCACCACGCC | TTTGGGGCTAGCCTATCGGCGGTAGATGTCC |
| *asrelE2*-down | CCCAAATTCGAATCAGTCCAGCAACACCGTC | TTTGGGTCCCGGATTTCTAGATGTACGGGGCGAAGTGATC |
| *asrelE2*-KO | AACTCACCGACGGGCGCT | AGCTGCGGGCATATCAGC |
| Ectopic Induction | | |
| P_myc2_*tetO2* | CCCAAATCTAGAGGATCGTCGGCACCGTCA | TTTGGGAAGCTTTTAATTAAGCGGCCGCATCGATGGATCGTGCTC ATTTCGG |
| *relE2*_WT_ | CCCAAAGGATCCGGCGGCTGTTGTCCCGGCTGTTGTGGCGGCGTGCCTTACACCGTGCGGT | TTTGGGGATATCCTATCGGCGGTAGATGTC |
| *relE2*_ΔR61L_ | CCCAAAGGATCCGGCGGCTGTTGTCCCGGCTGTTGTGGCGGCGTGCCTTACACCGTGCGGT | TTTGGGGATATCCTATCGGCGGTAGATGTCCGCGCGGTGATCGACGCGCAGGATCACTACCGTTGTGTGCTCGTCGTCAATCCGGTACAGCAGGAGGTACGTTCCGCGACGCG |
| *relE2*_ΔR81L,ΔY85F_ | CCCAAAGGATCCGGCGGCTGTTGTCCCGGCTGTTGTGGCGGCGTGCCTTACACCGTGCGGT | TTTGGGGATATCCTATCGGCGGAAGATGTCCGCGAGGTGATCGACGCGCAGGAT |
| *relB2*_WT_ | CCCAAAGCGGCCGCAATGCGGATACTGCCGATTTC | TTTGGGAAGCTTTCAGTGGGGGCGTCGC |
| *asrelE2-1*_WT_ | CCCAAAGCGGCCGCAGCAATACCGTCCACGCCA | TTTGGGAAGCTTGTGCCTTACACCGTGCGG |
| *asrelE2-2*_WT_ | CCCAAAGCGGCCGCAGCAATACCGTCCACGCCA | TTTGGGAAGCTTCAACTCACCGACGGGCGCT |
| *asrelE2-3*_WT_ | CCCAAAGCGGCCGCCTATCGGCGGTAGATGTCCG | TTTGGGAAGCTTGTGCCTTACACCGTGCGG |
| *his-relB2-his* | CCCAAAGCTAGCATGCGGATACTGCCGATTTC | TTTGGGCTCGAGGTGGGGGCGTCGCGGGA |
| *relE2-hsv* | CCCAAAGCATGCTCATGCCTTACACCGTGCGG | TTTGGGGCGGCCGCTCGGCGGTAGATGTCCG |
| *his-rnaseIII* | CCCAAAGCTAGCATGATCCGGTCACGACAAC | TTTGGGTTAATTAATTAGGCGGAGGTTTTGCC |
| *his-crp* | CCCAAAGCTAGCGTGGACGAGATCCTGGCC | TTTGGGTTAATTAATTACCTCGCTCGGCGGG |
| 5’-,3’-DIG Labeled Riboprobes | | |
| *relB2* | CAGUACAGCGUCUCCUGCAACGAUUCCCACUCGUCGGCGCCGACCAGAAC | |
| *relE2* | UUGCCCACCCGCAGGGGCUCGCGCGACAGAUCGCCGAACGCGAAUUCGAC | |
| asRelE2 | GUCGAAUUCGCGUUCGGCGAUCUGUCGCGCGAGCCCCUGCGGGUGGGCAA | |
| 5S rRNA | AUCGGCGCUGGCAGGCUUAGCUUCCGGGUUCGGAAUGGGACCGGGCGUUU | |
| T7 *In Vitro* Transcribed RNA | | |
| T7 *relE2* | CGAAATTAATACGACTCACTATAGGGAGAGTGCCTTACACCGTGCGG | CTATCGGCGGTAGATGTC |
| T7 asRelE2-3 | CGAAATTAATACGACTCACTATAGGGAGAGCAATACCGTCCACGCCA | GTGCCTTACACCGTGCGGTT |
| EMSA DNA probe primers and oligos | |  |
| WT P*_serC_*CBS | AGGGCTTGCATGTGAGCTT | GCCATCAGGGTAGTGAGG |
| WT P*_relBE2_*CBS | CCAGCAAACCTGAGACGC | ACCGCCATACTAGGCCGT |
| WT P*_asrelE2_*CBS | GCTGACGACGTCCTGTGC | TAACCCATTCTTTCAAGACC |
| Mut P*_serC_*CBS | AGGGCTTGCATGGTAGCTTGTTCACACTACGCGCCTGTGCCGGCAGGCTTTCGTTCCGCTCGAGTCGCCGCGGTTCGGTACCCCTCACTACCCTGATGGC | |
| Mut P*_relBE2_*CBS | CCAGCAAACCTGGAACGCCGCGCACAAAGTGCGAAACCACTGGAAGGTGAGCCCTAATTTAGGGCTGAGCAGGACCTGTATAACGGCCTAGTATGGCGGT | |
| Mut P*_asrelE2_*CBS | GCTGACGCAGTCCTGTGCACGGCGATGCGGTCTGGCAAGTGTCGGCGCTGCCAGTACCTCTCGATGCCAACAGGATCGTCGGTCTTGAAAGAATGGGTTA | |
| Transcriptional Reporter | | |
| WT P*_asrelE2_*CBS | CCCAAATCTAGACGAGCTGCGGGCATATCA | TTTGGGGGATCCAACCCATTCTTTCAAGACCG |
| Mut P*_asrelE2_*CBS | CCCAAATCTAGACGAGCTGCGGGCATATCAGCGCTGACGCAGTCCTGTGCACGGCGATGCG |  |
| *mcherry-ssrA* | CCCAAAGGATCCGGAGGAATCACTTCGCAATGGTCTCGAAGGGCGAG | TTTGGGAAGCTTTCAGGCGGCCAGGGCGTAGTCGCGCTGGTGCGAGTCGGCCTTGTACAGCTCGTCCATG |
| WT P*_relBE2_*CBS | CCCAAAAAGCTTAAGCACCAGCAAACCTGAG | TTTGGGACTAGTATCGATACCGCCATACTAGGCCGT |
| Mut P*_relBE2_*CBS | CCCAAAAAGCTTAAGCACCAGCAAACCTGGAACGCCGCGCACAAAGTGCG |  |
| *phgfp-ssrA* | CCCAAAATCGATGGAGGAATCACTTCGCAATGAGTAAAGGAGAAGAACTT | TTTGGGACTAGTTCAGGCGGCCAGGGCGTAGTCGCGCTGGTGCGAGTCGGCTTTGTATAGTTCATCCATGCC |
| 5’/3’ RACE |  |  |
| 5’ RNA Adaptor | GCUGAUGGCGAUGAAUGAACACUGCGUUUGCUGGCUUUGAUGAAA |  |
| 3’ Oligo(dT)_20_ |  | TGGCGATGAATGAACACTGCGTTTGCTGGCTTTGATGAAA(T)_20_ |
| 5’ RNA Adaptor F1 | CCCAAAGGATCCGCGTTTGCTGGCTTTGATGAAA |  |
| 3’ Oligo (dT)_20_ R1 |  | TTTGGGGATATCGCGTTTGCTGGCTTTGATGAA |
| 3’ *relE2* F1 | CCCAAAGGATCCGTGCCTTACACCGTGCGG |  |
| 5’ *relE2* R1 |  | TTTGGGGATATCTATCGGCGGTAGATGTCCG |
| 3’ *relB2* F1 | CCCAAAGGATCCATGCGGATACTGCCGATTT |  |
| 5’ *relB2* R1 |  | TTTGGGGATATCTCAGTGGGGGCGTCGCG |
| 3’ asRelE2 F1 | CCCAAAGGATCCGCAATACCGTCCACGCCA |  |
| 5’ asRelE2 R1 |  | TTTGGGGATATCGTGCCTTACACCGTGCGG |
| 5’ asRelE2 R2 |  | TTTGGGGATATCATGGCGGTATGCGGATAC |
| Sequencing Primer | TTTGCTGGCTTTGATGAAA |  |
| RT-qPCR |  |  |
| *cya* (*rv1264*) | CGTATCCGCAAGGGAGATTAG | CGTCAGGATCATCCACTCTG |
| *crp* | GTCCCGAAATCTCCGAACAG | GGCACATCGGTGAAGATGA |
| *relB2* | CACAGGACCAGATCACCATC | GATCGACTCCCTGATTCCG |
| *relE2* | TTACACCGTGCGGTTCAC | AACGCGAATTCGACCACT |
| asRelE2-3 | AACGCGAATTCGACCACT | TTACACCGTGCGGTTCAC |
| 16S rRNA | GGGTTAAGTCCCGCAACGAG | GCATGTGTGAAGCCCTGGAC |
| *Lrg-47* | AAAGGTCCACAGACAGCGTCACTCG | CAGGGGAGCATAATGGGTCTCTGC |
| *Igtp* | CCCATGGATTTAGTCACAAAGTTGC | GCTGGTGAGTCACTTTATTCCAGCC |
| *Cftr* | ACGCCCCTATGTCGACCAT | ACGACTATTATAGCTCCAATCACAATGA |
| *Atp6v1h* | AGCACTGCCTGGCCATACTTCCTG | TTCAACAGCAACACCGCTGCCAC |
| *Nramp1* | GCCACTGTGCTAGGTTTGCT | AATGGTGATCAGTACACCGC |
| *β-actin* | AGAGGGAAATCGTCGGTGAC | CAATAGTGATGACCTGGCCGT |
